# The application of desymmetric enantioselective reduction of cyclic 1,3-dicarbonyl compounds in the total synthesis of terpenoid and alkaloid natural products

**DOI:** 10.3762/bjoc.21.164

**Published:** 2025-10-14

**Authors:** Dong-Xing Tan, Fu-She Han

**Affiliations:** 1 Changchun Institute of Applied Chemistry, Chinese Academy of Sciences, 5625 Renmin Street, Changchun, Jilin 130022, China; 2 School of Applied Chemistry and Engineering, University of Science and Technology of China, Hefei, Anhui 230026, China

**Keywords:** alkaloids, cyclic 1,3-dicarbonyl compounds, desymmetrization, enantioselective reduction, terpenoids

## Abstract

The desymmetric enantioselective reduction of cyclic 1,3-dicarbonyl compounds is a powerful tool for the construction of ring systems bearing multiple stereocenters including all-carbon quaternary stereocenters, which are widely useful chiral building blocks for the total synthesis of structurally complex natural products. On the other hand, terpenoids and alkaloids, with their intricate and diverse skeletal frameworks as well as the broad range of biological activities, have long been a major focus for synthetic chemists. Over the past fifteen years, significant progress has been made in the total synthesis of complex terpenoid and alkaloid natural products by strategically applying desymmetric enantioselective reduction. Advance before 2016 in this area has been overviewed in an elegant review article. Since then, a series of more challenging terpenoid and alkaloid natural products have been synthesized utilizing a desymmetric enantioselective reduction strategy of cyclic 1,3-dicarbonyl compounds as a key transformation. This review will summarize the application of this strategy in the total synthesis of terpenoid and alkaloid natural products from the year 2016 to 2025. We first focus on the synthesis of several terpenoids and alkaloids through the desymmetric enantioselective reduction of five-membered cyclic 1,3-dicarbonyl compounds. Subsequently, the utilization of six-membered cyclic 1,3-dicarbonyl compounds for the synthesis of some terpenoids natural products is described.

## Introduction

Terpenoids and alkaloids are two major classes of highly important natural products because they usually exhibit diverse and important biological activities, such as antitumor, anti-inflammatory, and antiarrhythmic effects etc., and show potential to be developed into drug candidates or novel medications for treating human diseases [1–4]. However, their scarcity in nature limits further research into their biological activities. Total synthesis is an important device to address the shortage of natural product sources. Nevertheless, the asymmetric total synthesis of terpenoid and alkaloid natural products presents significant challenges due to their complex and diverse ring systems and the presence of multiple stereocenters, including all-carbon quaternary stereocenters. Consequently, the development of novel methods and strategies to achieve efficient asymmetric total synthesis of complex terpenoid and alkaloid natural products has drawn considerable attention from synthetic chemists.

**Figure 1:**
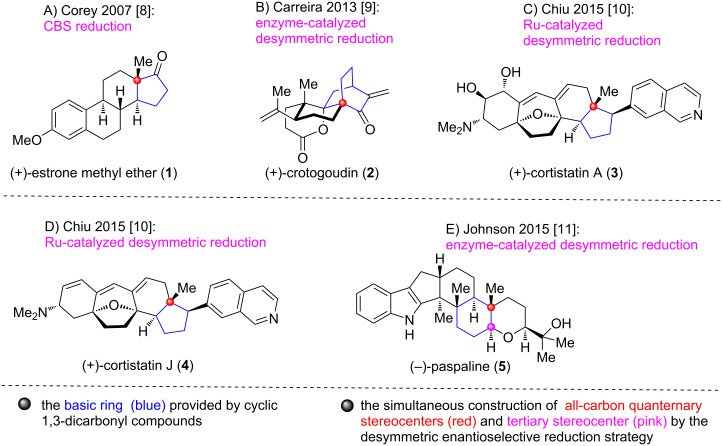
Several representative terpenoid and alkaloid natural products synthesized by applying desymmetric enantioselective reduction strategy of cyclic 1,3-dicarbonyl compounds before 2016.

Over the past decades, the development of desymmetric enantioselective reduction strategy of cyclic 1,3-dicarbonyl compounds has invigorated the field of terpenoid and alkaloid natural products synthesis because of its multiple advantages. Specifically, such strategy allows for an efficient construction of multiple chiral centers by employing easily accessible or commercially available symmetric cyclic prochiral dicarbonyl substrates. In addition, various approaches could be used for the desymmetrization reactions such as enzyme catalytic-, organocatalyst-, and transition-metal-catalyzed reductions [5–7]. Advance about the synthesis of several terpenoid and alkaloid natural products (**1**–**5**, Figure 1) [8–11] has been achieved in this area as indicated by an elegant review in 2016 [12], which will not be discussed in this review.

**Figure 2:**
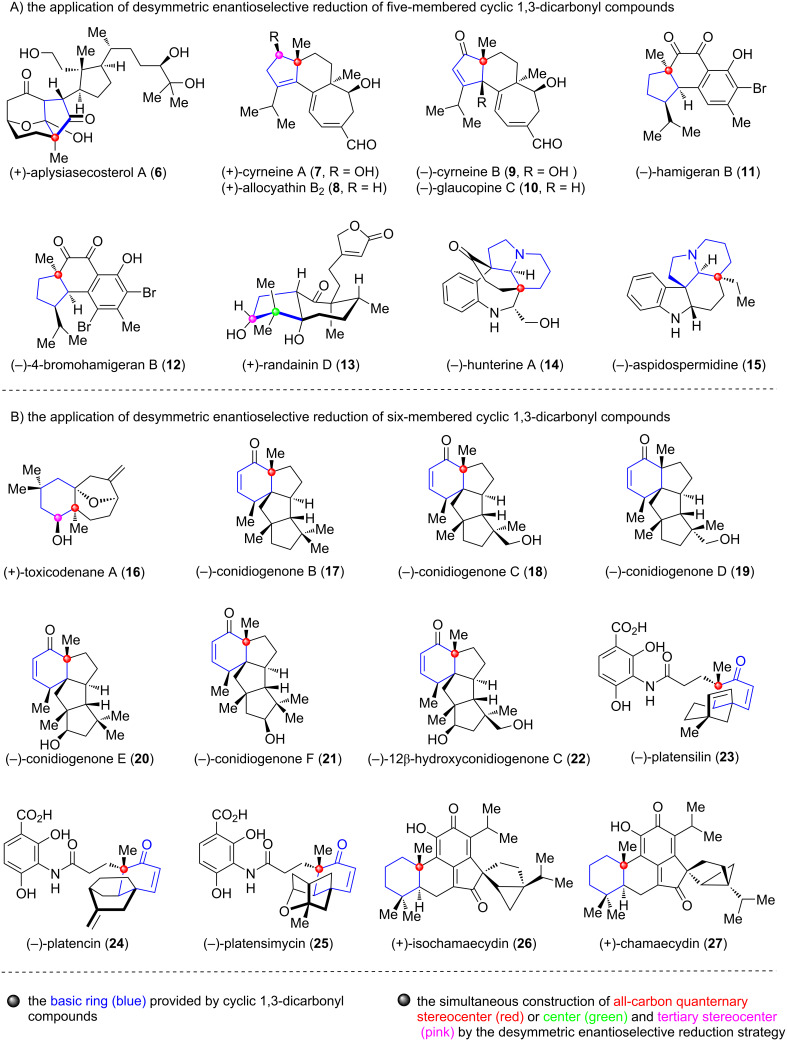
Selected terpenoid and alkaloid natural products synthesized by applying desymmetric enantioselective reduction strategy of cyclic 1,3-dicarbonyl compounds in 2016–2025.

Although only a limited number of terpenoids and alkaloids were synthesized by applying the desymmetric enantioselective reduction strategy of cyclic 1,3-dicarbonyl compounds before 2016, their success has provided a reference for the subsequent synthesis of other types of terpenoid and alkaloid natural products. In fact, over the past ten years, the number of terpenoid and alkaloid natural products synthesized utilizing this strategy has increased significantly compared to the period before 2016. However, there is currently a lack of systematic summary regarding the application of this strategy in the total synthesis of terpenoids and alkaloids over this time span. This review will provide a comprehensive overview of the recent advances (2016–2025), specially, we first summarize chronologically the application of desymmetric enantioselective reduction of five-membered cyclic 1,3-dicarbonyl compounds in the synthesis of terpenoid and alkaloid natural products **6**–**15** (Figure 2A). Subsequently, the advances in the utilization of six-membered cyclic 1,3-dicarbonyl compounds for the synthesis of terpenoid natural products **16**–**27** will be introduced in chronological order (Figure 2B). After that, a brief outlook of the desymmetric enantioselective reduction strategy of cyclic 1,3-dicarbonyl compounds in the total synthesis of natural products will be discussed. We hope this review can stimulate the methodology development and application of this strategy toward further improving the synthetic efficiency of natural product synthesis.

## Review

### Total synthesis of terpenoid and alkaloid natural products through the desymmetric enantioselective reduction of five-membered cyclic 1,3-dicarbonyl compounds

#### Total synthesis of (+)-aplysiasecosterol A

(+)-Aplysiasecosterol A (**6**) is a secosteroid that was isolated from the sea hare *Aplysia kurodai* by Kita and Kigoshi in 2015 [13]. Due to its natural scarcity, the biological activity has not been explored. In 2018, Li and co-workers accomplished the first asymmetric total synthesis of (+)-aplysiasecosterol A (**6**) by employing a desymmetric enantioselective reduction strategy of 1,3-cyclopentanedione derivative as the key transformation [14].

**Scheme 1:**
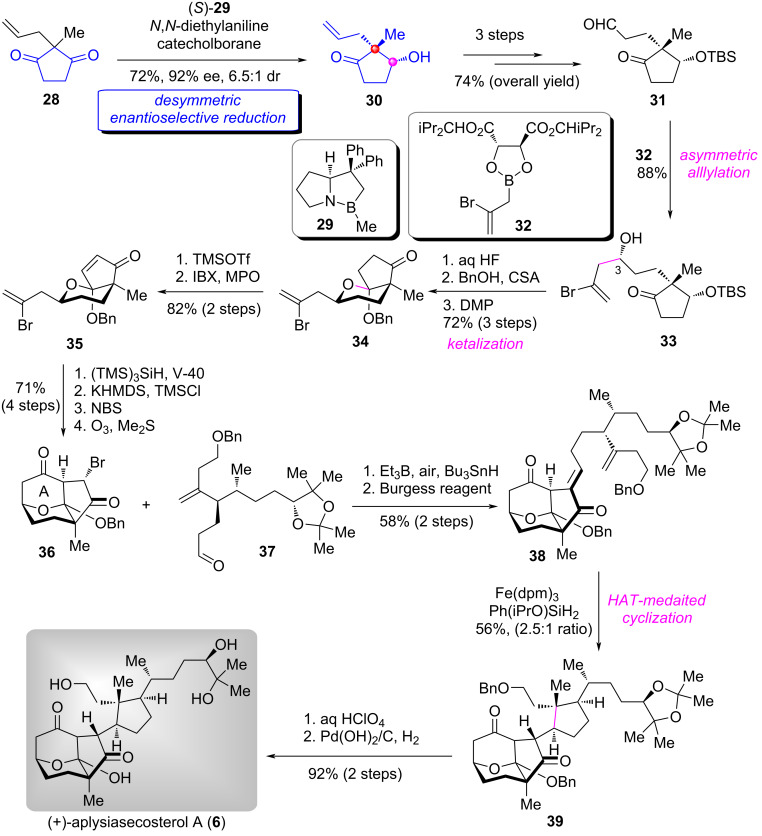
The total synthesis of (+)-aplysiasecosterol A (**6**) by Li [14].

Their synthesis features a highly efficient desymmetric enantioselective reduction of diketone **28** for preparing alcohol **30** under the CBS conditions [8] with (*S*)-**29** as the catalyst (Scheme 1) [14]. Notably, this reaction could be performed on multiple gram scales with satisfactory yield (72%) and ee value (92%). Protection of the alcohol group in **30** with TBSCl followed by modification of the terminal double bond afforded ketoaldehyde **31**. The 2-bromoallylation [15] of **31** with boronic ester **32** stereoselectively constructed the C3–OH group to give homoallylic alcohol **33**. Next, a successive manipulation by removal of TBS group, CSA-catalyzed ketalization, and DMP oxidation of the secondary alcohol to a ketone allowed for rapid construction of bicyclic ketone **34** in high overall yield. The oxidative dehydrogenation of **34** gave α,β-unsaturated bicyclic ketone **35** smoothly. Sequential A-ring construction and functional group modifications of **35** produced the diketone **36**. Subsequently, **36** and aldehyde **37**, which was prepared with 9 steps from commercially available (+)-citronellol, underwent a Reformatsky-type radical addition under the conditions of Et_3_B/air/Bu_3_SnH to deliver aldol product [16]. Dehydration of the secondary alcohol gave (*E*)-**38**. The HAT radical cyclization [17] of **38** in the presence of Fe(dpm)_3_/Ph(iPrO)SiH_2_ proceeded smoothly to furnish the tetracyclic product **39** in 56% yield with 2.5:1 ratio. Finally, removal of the acetonide and the Bn protecting group completed the total synthesis of (+)-aplysiasecosterol A (**6**).

#### Total synthesis of (+)-cyrneine A, (−)-cyrneine B, (−)-glaucopine C, and (+)-allocyathin B_2_

The cyrneine diterpenoids represent an important subfamily of cyathane diterpenoids which possess a common 5-6-7 fused tricarbocyclic core with two all-carbon quaternary stereocenters. Many impressive syntheses have been reported to date [18–30]. In 2018, the group of Han accomplished the total synthesis of (+)-cyrneine A (**7**), (−)-cyrneine B (**9**), (−)-glaucopine C (**10**), and (+)-allocyathin B_2_ (**8**) by a collective manner [31]. In their synthetic route, an enzyme-catalyzed desymmetric enantioselective reduction of 1,3-cyclopentanedione derivative was adopted as one of the key reactions, which facilitated the construction of the five-membered ring bearing an all-carbon quaternary center as the key chiral building block.

**Scheme 2:**
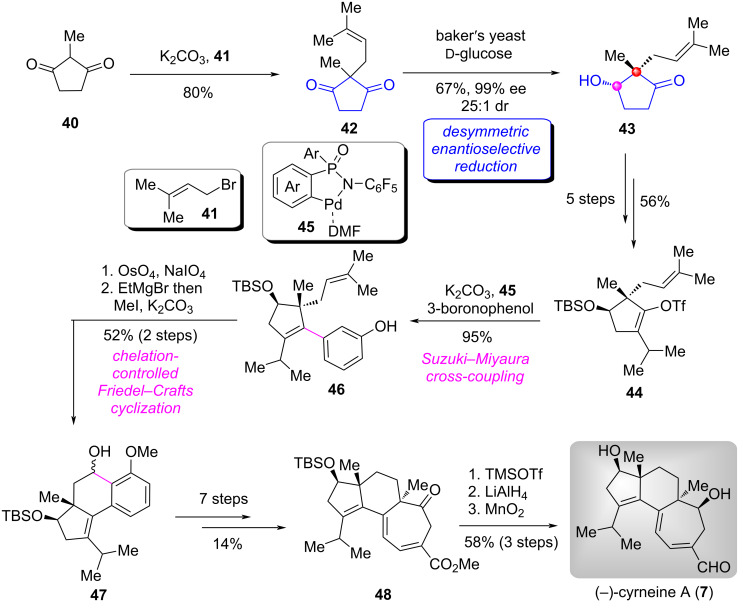
The total synthesis of (−)-cyrneine A by Han [31].

Their synthesis began with 1,3-dione **40** (Scheme 2) [31–32], allylation of this substrate with allylic bromide **41** afforded the 1,3-cyclopentanedione derivative **42**. Next, the baker′s yeast-catalyzed desymmetric enantioselective reduction of **42** gave the α-hydroxyketone **43** in satisfactory yield and excellent stereoselectivity and diastereoselectivity on a decagram scale [33–34]. Functional group modifications and transformations of **43** produced hydroxyketone **44**. Due to the steric hindrance of this substrate, the subsequent Suzuki cross coupling reaction with 3-boronophenol proceeded in low yield. To address this issue, Han′s group employed a novel palladacycle catalyst **45**, previously developed by their group [35–37]. This catalytic system efficiently overcame the challenge and furnished the coupling product **46** in high yield. Oxidative cleavage of the double bond in **46** followed by Mg(II)-mediated chelation-controlled Friedel–Crafts cyclization delivered secondary alcohol **47**, which was elaborated to ketone **48** via a seven-step transformation. Finally, removal of the TBS group in **48** followed by a sequential reduction and selective oxidation of allylic primary alcohol achieved the total synthesis of (−)-cyrneine A (**7**).

**Scheme 3:**
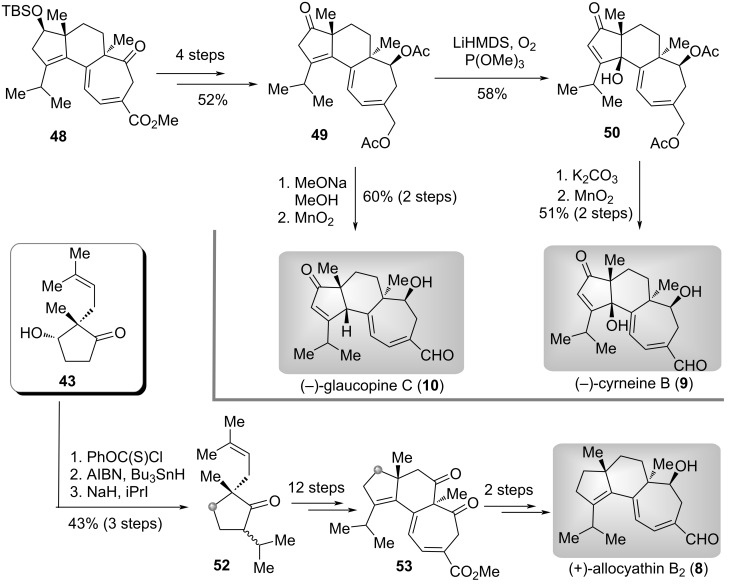
The total syntheses of three cyrneine diterpenoids by Han [31–32].

The authors then moved forward to the synthesis of other target molecules (Scheme 3) [31–32]. Oxidation state adjustment of **48** led to the ketone **49**. Starting from this common intermediate, firstly, base-promoted double bond migration and oxidation at the γ-position gave tertiary alcohol **50**. Deprotection of acetyl in **50** followed by selective oxidation delivered (−)-cyrneine B (**9**). Secondly, a based-mediated concomitant double bond migration and deacetylation, and selective oxidation of allylic primary alcohol accomplished the total synthesis of (−)-glaucopine C (**10**). On the other hand, Barton–McCombie deoxygenation and isopropylation of **43** produced ketone **52**. Subsequently, by employing the same procedures for the synthesis of (−)-cyrneine A (**7**), the synthesis of (+)-allocyathin B_2_ (**8**) could also be achieved smoothly from **52** by utilizing diketone **53** as the intermediate. The diverse syntheses of these terpenoids enabled by the desymmetric enantioselective reduction of cyclic 1,3-dicarbonyl compounds demonstrated the significance of the desymmetric reduction strategy in the synthesis of structurally complex natural products.

#### Total synthesis of (−)-hamigeran B and (−)-4-bromohamigeran B

(−)-Hamigeran B (**11**) and (−)-4-bromohamigeran B (**12**) were isolated from the sponge *Hamigera tarangaensis*, and show cytotoxicity against various tumor cells. Notably, compound **11** exhibits 100% inhibition against herpes and polio viruses without significant host cell cytotoxicity [38]. Since their isolation, the synthesis of **11** and **12** have been reported by many groups [39–50]. For an efficient synthesis of these two natural products, Han and co-workers [51] adopted an alternative route utilizing the desymmetric enantioselective reduction strategy of a 1,3-cyclopentanedione derivative as the key transformation. Both (−)-hamigeran B (**11**) and (−)-4-bromohamigeran B (**12**) were synthesized by a divergent manner with a longest linear sequence of 14 steps from the known symmetrical diketone **28** (see Scheme 1) [14]. More importantly, this route allowed for the synthesis of target compounds at 100 mg scale in a single batch.

**Scheme 4:**
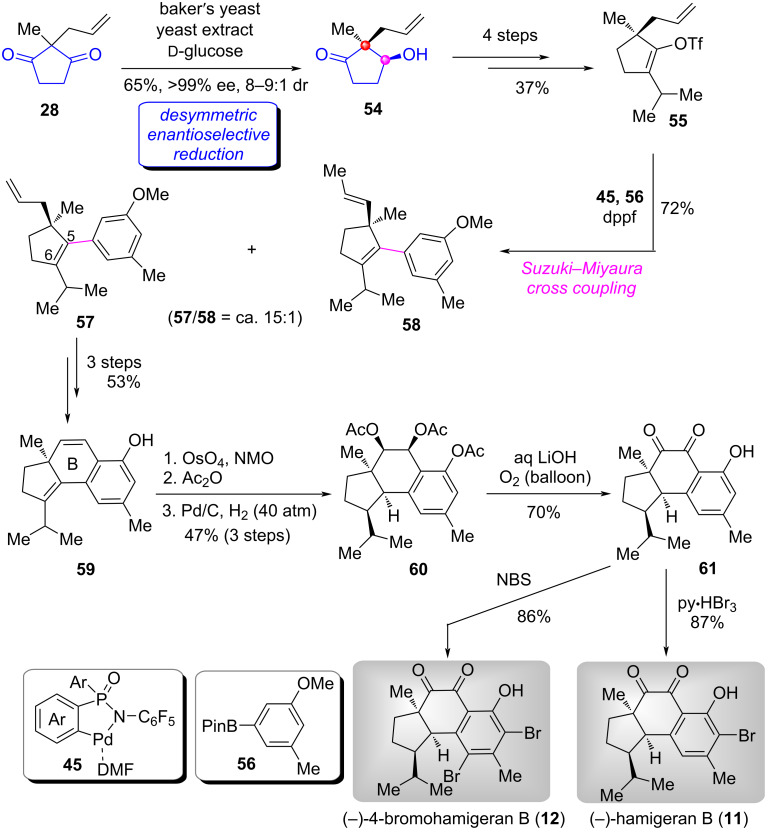
The total synthesis of (−)-hamigeran B and (−)-4-bromohamigeran B by Han [51].

As shown in Scheme 4 [51], the enzyme-catalyzed desymmetric enantioselective reduction of **28**, afforded hydroxyketone **54** in 65% yield with >99% ee and 8–9:1 dr on multigram scale [34]. Functional group transformations of **54** in four steps produced sterically hindered allyl triflate **55**. By employing the palladacycle catalyst **45**-catalyzed Suzuki cross-coupling reaction of sterically hindered substrates developed by Han [35–37], the coupled product **57** was obtained in a satisfactory yield (72%) from **55** and pinacol boronate **56,** along with trace amounts of the double bond migrated side product **58** (**57**:**58** = ca. 15:1). Demethylation of **57** to phenolic intermediate followed by the construction of the B ring generated tricyclic core **59**. Subsequently, dihydroxylation of the doubled bond in the central six-membered ring using OsO_4_/NMO gave diol, which was then subjected to acetylation of the two hydroxy groups and hydrogenation of C5=C6 double bond to afford triacetate **60** as a single diastereoisomer. Base-promoted hydrolysis and concomitant oxidation under oxygen atmosphere gave vicinal diketone **61**. Finally, the introduction of mono-bromo and di-bromo atoms achieved the total synthesis of (−)-hamigeran B (**11**) and (−)-4-bromohamigeran B (**12**), respectively.

#### Total synthesis of (+)-randainin D

(+)-Randainin D (**13**) is a representative member of a class of structurally intriguing diterpenoids containing a *trans*-hydroazulenone core and a C9-butenolide moiety isolated from *Callicarpa randaiensis* [52]. It exhibits inhibition of elastase release and superoxide-anion generation. In 2024, Baudoin and co-workers presented an efficient route for the first total synthesis (+)-randainin D (**13**) by utilizing an early-staged Ru-catalyzed desymmetric enantioselective reduction as the key transformation [53].

**Scheme 5:**
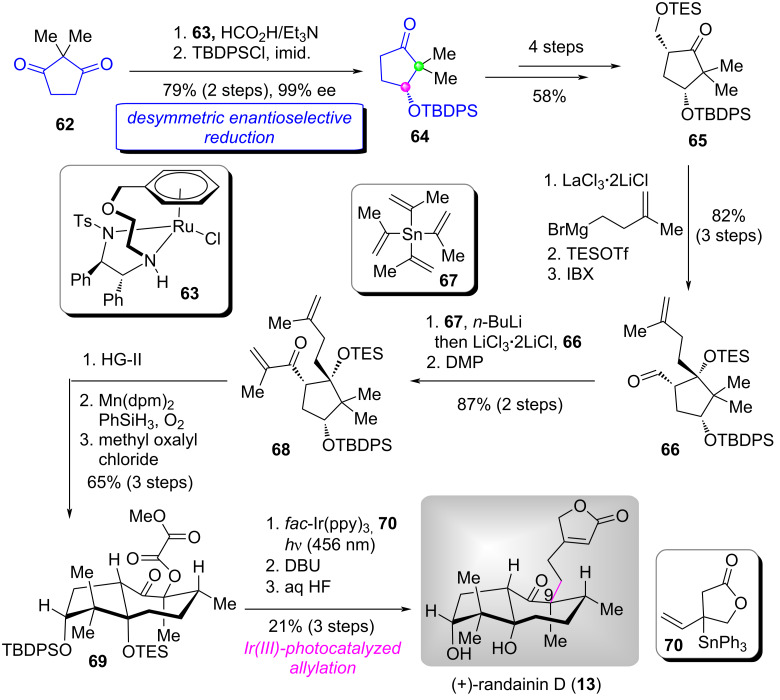
The total synthesis of (+)-randainin D by Baudoin [53].

As shown in Scheme 5 [53], based on the reported method for asymmetric transfer hydrogenation of commercially available cyclopentadione **62** [54], the authors adapted an efficient method for the desymmetric enantioselective reduction of **62** using commercially available (*R*,*R*)-Ts-DENEB (**63**) as the catalyst and formic acid as the hydrogen donor, securing the desired secondary alcohol product in excellent enantioselectivity (99% ee). Protection of the alcohol group with TBDPSCl gave silyl ether **64** in high yield (79% for 2 steps). Subsequently, successive four manipulations including dehydrogenation, Morita–Baylis–Hillman reaction, protection of the resultant primary alcohol, and hydrogenation afforded ketone **65**. The LaCl_3_·LiCl-promoted addition of **65** with Grignard reagent followed by TES protection of the resulting secondary alcohol, regioselective deprotection of the TES group and in situ oxidation provided aldehyde **66**. Next, **66** underwent the 1,2-addition of isopropenyllithium reagent (prepared from *n*-BuLi/tetraisopropenyltin (**67**) [55]) and DMP oxidation to afford ketone **68**. A three-step transformation including RCM, Mukaiyama hydration, and esterification, **68** was converted to methyl oxalate **69**. Irradiation of the reaction mixture **69** and lactone **70** at 456 nm with *fac*-Ir(ppy)_3_ as the photocatalyst furnished a mixture of isomeric olefins. Finally, DBU-promoted the isomeric olefins conjugation and removal of the two silyl ether completed the first total synthesis of (+)-randainin D (**13**).

#### Total synthesis of (−)-hunterine A and (−)-aspidospermidine

(−)-Hunterine A (**14**) and (−)-aspidospermidine (**15**) are monoterpene indole alkaloids, which were isolated from *Hunteria zeylanica* and *Apocynaceae* plants, respectively [56–57]. Structurally, these two natural products both contain the fused polycyclic skeleton core bearing four consecutive stereocenters, two of which are all-carbon quaternary stereocenters. Such a skeletally extraordinary structure poses significant challenges for total synthesis. In 2024, Stoltz and co-workers finished the divergent enantioselective total synthesis of (−)-hunterine A and (−)-aspidospermidine by utilizing a Ru-catalyzed desymmetric enantioselective reduction of bicyclic 1,3-diketone **72** for rapidly accessing the 5/5 bicyclic core skeleton containing two desired stereocenters [58]. The application of this strategy accelerated the synthesis of these two complex alkaloids within a longest linear sequence of 16 and 14 steps, respectively.

**Scheme 6:**
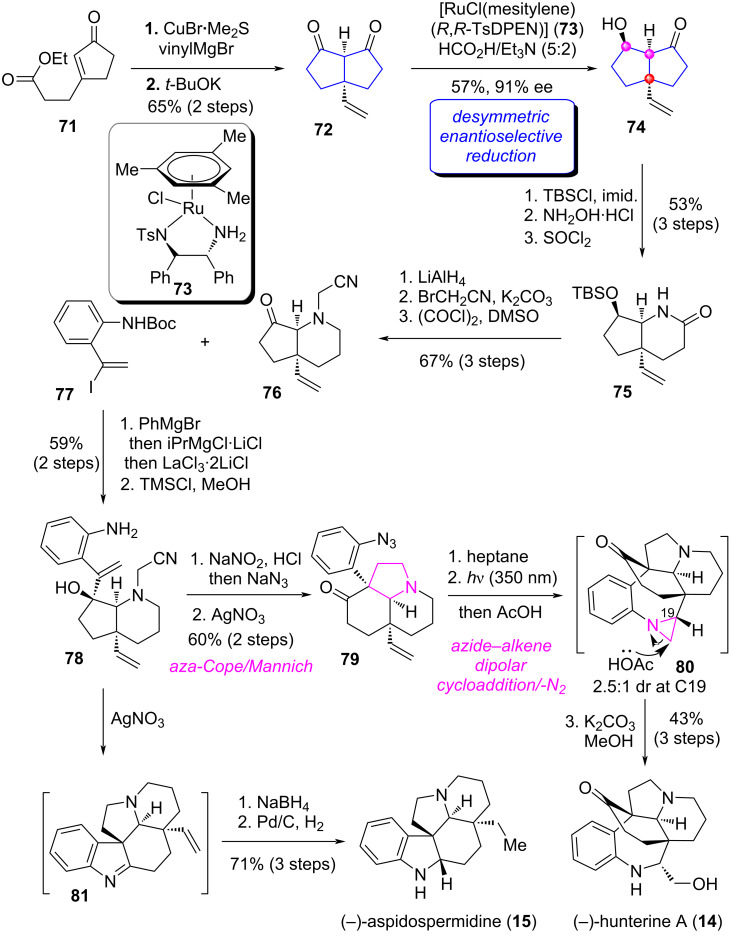
The total synthesis of (−)-hunterine A and (−)-aspidospermidine by Stoltz [58].

As shown in Scheme 6 [58], the Cu-catalyzed 1,4-conjugated addition of **71** [59] with vinylMgBr followed by intramolecular Claisen condensation furnished bicyclic diketone **72** on a gram scale. Notably, for such a bicyclic substrate, it is quite challenging to achieve desymmetric enantioselective reduction because the catalyst has difficulty identifying its *Re*- and *Si*-faces. After screening several reduction strategies, the authors identified a set of Ru-catalyzed transfer hydrogenation conditions [60–61] utilizing **73** as the catalyst that could be used for the desymmetric enantioselective reduction of **72**, affording the hydroxyketone **74** in 57% yield with 91% ee. Protection of the secondary alcohol in **74** followed by Beckmann rearrangement led to lactam **75**. Oxidation state modifications and functional group transformations of **75** afforded ketone **76**. Next, the 1,2-addition of **76** with vinyl iodine **77** and subsequent deprotection produced tertiary alcohol **78**. Starting from this common intermediate, on the one hand, through successive manipulations by diazotization and in situ azide substitution, AgNO_3_-mediated aza-Cope/Mannich [62] reaction delivered ketone **79**. Subsequently, a three-step operation including azide–alkene dipolar cycloaddition, irradiation of the resulting triazoline to aziridine **80** and in situ ring opening followed by deacetylation achieved the first total synthesis of (−)-hunterine A (**14**). On the other hand, aza-Cope/Mannich reaction of **78** produced imine intermediate **81**. Reduction and hydrogenation of **81** furnished the total synthesis of (−)-aspidospermidine (**15**).

### Total synthesis of terpenoid natural products through the desymmetric enantioselective reduction of six-membered cyclic 1,3-dicarbonyl compounds

#### Total synthesis of (+)-toxicodenane A

**Scheme 7:**
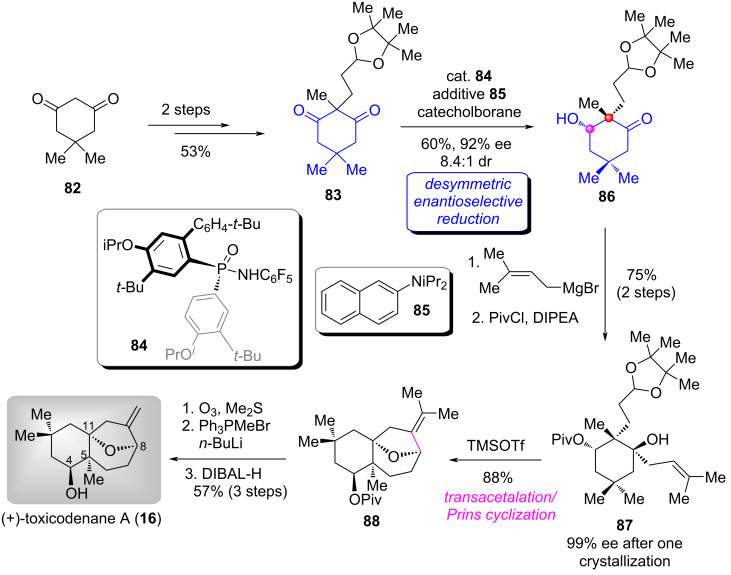
The total synthesis of (+)-toxicodenane A by Han [65–66].

The skeletally new sesquiterpenoids, toxicodenanes A–C and E (see representative structure **16** in Scheme 7) were isolated from *Toxicodendron vernicifluum* in 2013 to 2015 [63–64], whose structures feature the all-carbon bicyclic skeleton and four to seven contiguous stereocenters, posing significant challenges to their synthesis. Additionally, there is a lack of corresponding pharmacological activity studies. In 2021, the group of Han completed the first enantioselective total synthesis of (+)-toxicodenane A (**16**) and determined its absolute configuration by employing an early-stage desymmetric enantioselective reduction of a 1,3-cyclohexanedione derivative as the key transformation [65]. The application of this strategy markedly accelerated the synthesis of the complex molecule within a longest linear sequence of 9 steps from the commercially available material.

The synthesis began with the commercially available 1,3-cyclohexanedione **82** (Scheme 7) [65–66]. Accordingly, the α-dialkylation of **82** gave diketone **83**. Based on the conditions developed by the authors [67], namely, using *P*-stereogenic phosphinamide **84** as the catalyst and base **85** as the additive, the desymmetric enantioselective reduction of **83** proceeded smoothly to deliver the hydroxyketone **86** in 60% yield with 92% ee and 8.4:1 dr. A secondary hydroxy-directed Grignard reagent addition of **86** followed by selective protection, generated alcohol ester **87**. After one recrystallization, the ee value of **87** could be increased to 99%. Subsequently, TMSOTf-promoted transacetalation and in situ Prins cyclization produced the oxa-bridged product **88**. Finally, ozonolysis of **88** followed by Wittig reaction of the resultant ketone and subsequent reduction accomplished the total synthesis of (+)-toxicodenane A (**16**). The authors eventually confirmed that the absolute configuration of (+)-**16** was 4*S*,5*S*,8*R*,11*R* based on the X-ray single-crystal analysis of its corresponding *p*-bromobenzoic ester (not shown).

#### Total synthesis of (−)-conidiogenones B–F and (−)-12β-hydroxyconidiogenone C

Conidiogenones are unique diterpenoids which possess a highly congested 6/5/5/5-fused framework with four all-carbon quaternary centers isolated from *Penicillium cylopium* and exhibit various biological properties [68]. The intriguing structure and interesting biological properties have attracted continued synthetic attention [69–71]. In a 2023 report, the group of Lee and Han adopted an early-stage desymmetric enantioselective reduction of 1,3-cyclohexanedione derivative **89** as the key transformation [72]. Both (−)-conidiogenones B–F (**17**–**21**) and (−)-12β-hydroxyconidiogenone C (**22**) were synthesized in a divergent manner.

**Scheme 8:**
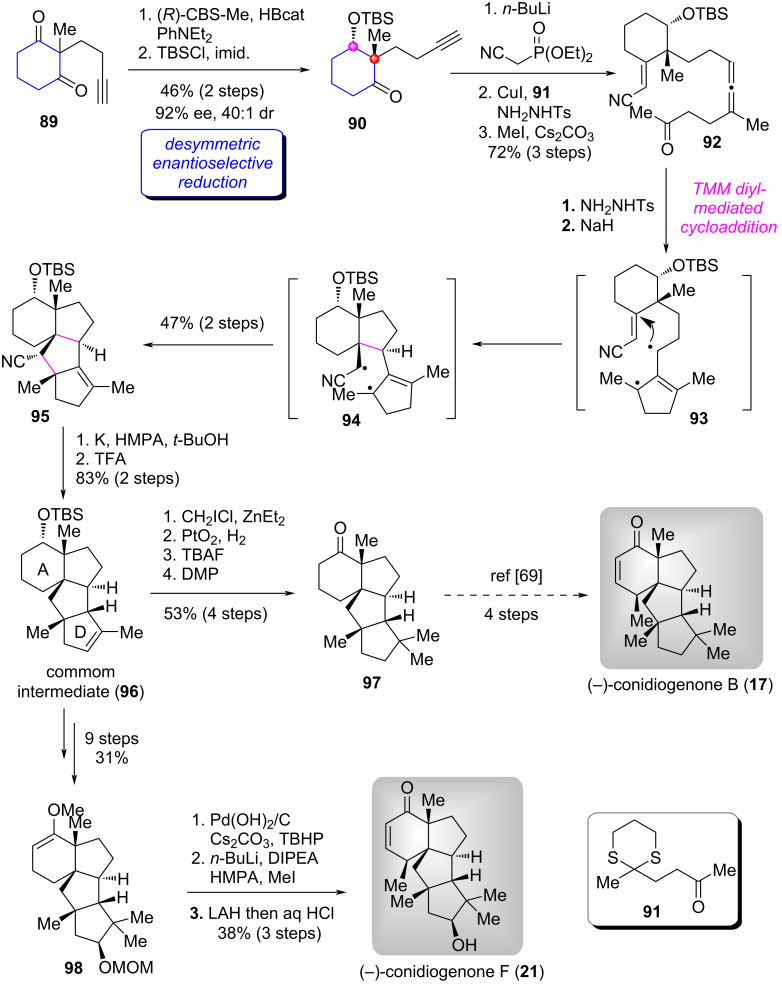
The formal total synthesis of (−)-conidiogeone B and total synthesis of (−)-conidiogeone F by Lee and Han [72].

Their synthetic route began with the known terminal alkyne cyclohexanedione **89** [73]. As illustrated in Scheme 8 [72], the easily prepared substrate underwent the CBS reduction conditions and protection of the resulting secondary alcohol with TBSCl to afforded silyl ether ketone **90** in 46% yield (two steps, dr = 40:1, 92% ee) [8]. Horner–Wadsworth–Emmons (HWE) reaction of **90** followed by Cu-carbene migratory insertion [74] with ketone **91** and deprotection of the dithiane group delivered alleneketone **92**. Sequential treatment of **92** with TsNHNH_2_ and NaH, the trimethylenemethane (TMM) diyl-mediated cycloaddition via intermediates **93** and **94** proceeded uneventfully to form the tetracyclic product **95**. Next, reductive decyanation [75] and double bond migration of **95** produced common intermediate **96**, which was elaborated to ketone **97** [69] via four functional group manipulations, thereby achieving the formal total synthesis of (−)-conidiogenone B (**17**). On the other hand, functionalization and derivatization were performed on the A- and D-rings of **96**, respectively, delivering the methyl enol ether **98**. Finally, allylic oxidation [76], α-methylation and reduction of **98** followed by hydrolysis accomplished the first total synthsis of (−)-conidiogenone F (**21**).

**Scheme 9:**
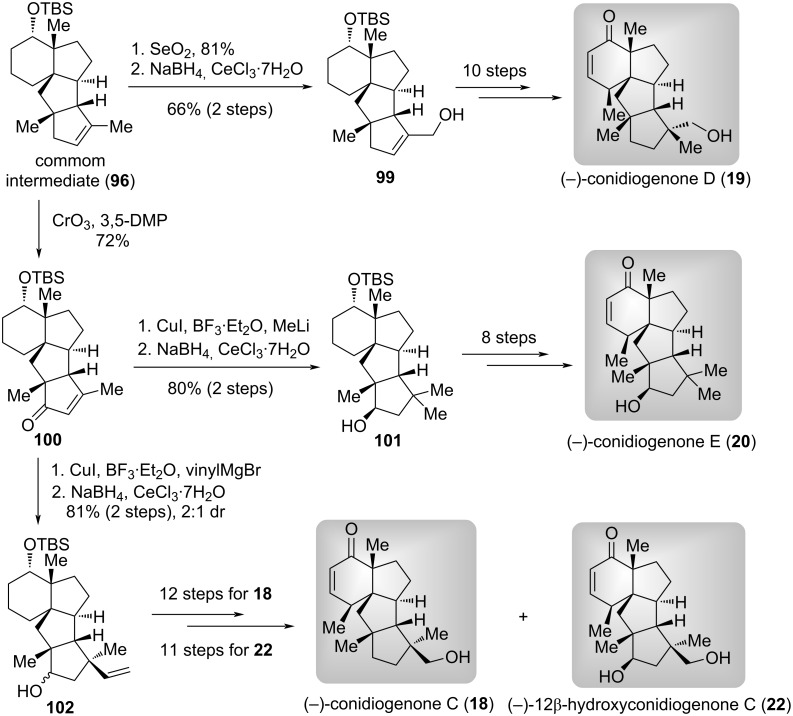
The total syntheses of four conidiogenones natural products by Lee and Han [72].

After achieving the aforementioned success, the authors continued to utilize **96** as the common intermediate to synthesize other target natural products (Scheme 9) [72]. Firstly, the allylic oxidation and Luche reduction of **96** afforded primary alcohol **99**, which was elaborated to (−)-conidiogenone D (**19**) via ten functional group manipulations. Secondly, the allylic oxidation of **96** with CrO_3_/3,5-DMP produced ketone **100**. The 1,4-conjugate addition of **100** with MeI followed by Luche reduction provided the secondary alcohol **101**. A-ring modifications in **101** completed the first total synthesis of (−)-conidiogenone E (**20**). One the other hand, a two-step transformation involving the 1,4-conjugate addition utilizing vinylMgBr and Luche reduction, **100** was converted to secondary alcohol **102** (dr = 2:1). Finally, functional group modifications of A- and D-rings furnished the total syntheses of (−)-conidiogenone C (**18**) and (−)-12β-hydroxyconidiogenone C (**22**), respectively.

#### Total syntheses of (−)-platensilin, (−)-platencin, and (−)-platensimycin

(−)-Platensilin (**23**), (−)-platencin (**24**), and (−)-platensimycin (**25**) are structurally unique meroterpenoids containing rigid bicycle [3.2.1] or [2.2.2]-octane cage core. They present promising drug leads for both antidiabetic and antibacterial therapies. The intriguing structural features and potential bioactivities have stimulated tremendous synthetic efforts [77–81]. In 2024, the group of Lou and Xu presented a unified and efficient route for the syntheses of (−)-platensilin, (−)-platencin, and (−)-platensimycin by utilizing a desymmetric enantioselective reduction of 1,3-cyclohexanedione derivative strategy [82].

**Scheme 10:**
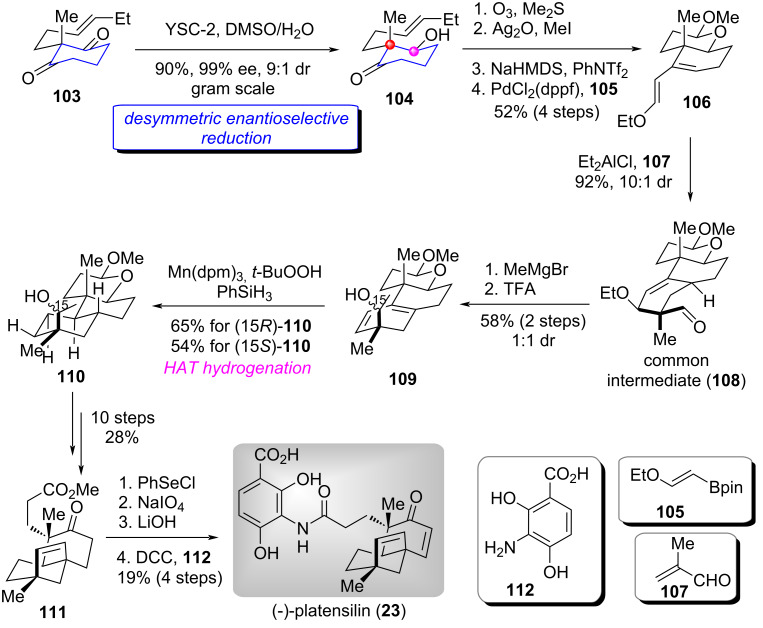
The total synthesis of (−)-platensilin by Lou and Xu [82].

The synthesis of (−)-platensilin (**23**) is shown in Scheme 10 [82]. Brewer′s yeast (YSC-2)-promoted desymmetric enantioselective reduction of 1,3-cyclohexanedione derivative **103** proceeded smoothly to produce hydroxyketone **104** in excellent diastereoselectivity and enantioselectivity [9,11]. Ozonolysis of the double bond in **104** followed by Purdie methylation with Ag_2_O/MeI, base-mediated vinyl triflation, and Pd-catalyzed Suzuki–Miyaura cross coupling with pinacol boronate **105** delivered diene **106**. Next, The Et_2_AlCl-catalyzed intermolecular Diels–Alder reaction of **106** with methacrolein **107** afforded the common intermediate **108** in high yield. Sequential Grignard reagent addition and acid-promoted ethoxy elimination provided the separable planar diene **109** (dr = 1:1), which underwent a Mn-catalyzed HAT hydrogenation to give (15*R*)-**110** and (15*S*)-**110** in 65% and 54% yield, respectively. Subsequently, ten functional group manipulations of the diastereomeric mixture **110** produced ketoester **111**. Finally, the introduction of conjugated double bond in **111** followed by hydrolysis of the methyl ester to carboxylic acid and DCC-mediated condensation with **112** accomplished the total synthesis of (−)-platensilin (**23**).

**Scheme 11:**
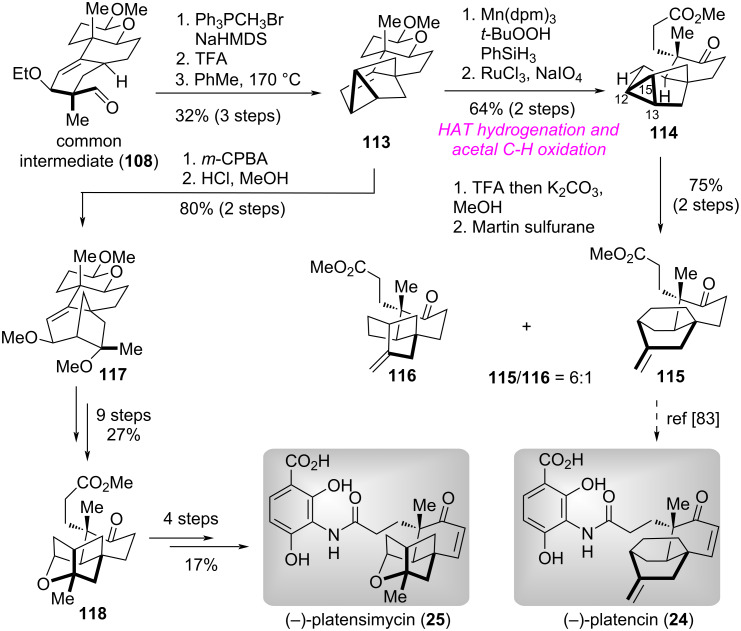
The total synthesis of (−)-platencin and (−)-platensimycin by Lou and Xu [82].

Based on the aforementioned successful work, the authors focused on the synthesis of (−)-platencin (**24**) and (−)-platensimycin (**25**) (Scheme 11) [82]. Accordingly, Wittig reaction of **108** followed by 1,4-elimination and intramolecular Diels–Alder reaction generated tricyclo[3.2.1.0^2,7^]-octene **113**. A two-step transformation including HAT hydrogenation and acetal C–H oxidation with RuCl_3_/NaIO_4_, **113** was converted into ketoester **114**. The TFA-mediated C13–C15 bond cleavage of **114** proceed smoothly to give ring-opening products, which underwent dehydration with Martin′s sulfurane to afford the known intermediate **115** [83] and terminal alkene **116** (C12–C13 bond-cleaved byproduct). Thus, the formal total synthesis of (−)-platencin (**24**) was achieved. On the other hand, epoxidation of **113** followed by acid-mediated regioselective ring-opening and in situ allylic substitution with MeOH produced [3.2.1] bridged ring product **117**, which was transformed into ketone **118** via nine functional group manipulations. Finally, by employing the same reaction procedures as those utilized in the total synthesis of (−)-platensilin (**23**), the authors accomplished the total synthesis of (−)-platensimycin (**25**).

#### Total synthesis of (+)-isochamaecydin and (+)-chamaecydin

(+)-Isochamaecydin (**26**) and (+)-chamaecydin (**27**) are represented members of the cryptoquinonemethides isolated from the seed of *chamaecyparis obtusa* Endl. genus [84]. This two compounds possess the unprecedented spiroannulated 6/6/6/5/5/3 (A/B/C/D/E/F) hexacarbocyclic motif and exhibit significant antifeedant activity against the pest insect *Spodoptera litura* [85]. In a very recent report, Han and co-workers accomplished the first catalytic asymmetric total syntheses of **26** and **27** via a modular and convergent strategy [86]. The key to their successful synthesis depended on the method application of a desymmetric enantioselective reduction of a 1,3-cyclohexanedione derivative as the key transformation.

**Scheme 12:**
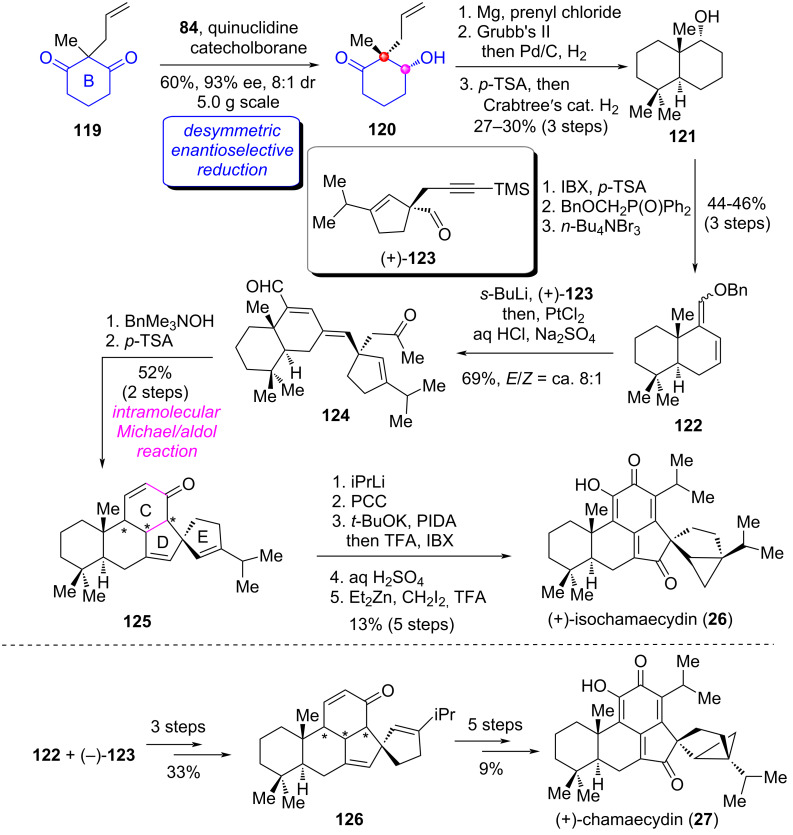
The total synthesis of (+)-isochamaecydin and (+)-chamaecydin by Han [86].

Their synthesis began with the known 1,3-cyclohexanedione **119** (Scheme 12) [86]. Initially, for the construction of the crucial B ring bearing an all-carbon quaternary stereocenter, the authors employed a method they had previously reported [35,67]. Namely, the *P*-stereogenic phosphinamide (**84**, see Scheme 7)-catalyzed desymmetric enantioselective reduction of **119**. Hydroxyketone **120** could be acquired in 60% yield with 93% ee. Subsequently, the Grignard addition of the ketone in **120** provided diene intermediate, which was converted to bicyclic secondary alcohol **121** via RCM reaction/hydrogenation and dehydration/hydrogenation. Oxidation states adjustment and functional group transformations of **121** generated bromodiene **122**. Next, the metal–halogen exchange/intermolecular addition of **122** with aldehyde (+)-**123** and in situ PtCl_2_-promoted hydrolysis and hydration gave tricyclic product **124**. The BnMe_3_NOH-mediated intramolecular Michael/aldol cascade reaction of **124** constructed the C/D rings, followed by dehydration to afford pentacyclic product **125**. Finally, a five-step operation including the 1,2-addition of **125** with iPrLi, PCC oxidation of the resulting tertiary alcohol, oxidative aromatization and in situ neutralization/oxidation, acid-mediated 1,6-addition with H_2_O, and stereoselective cyclopropanation accomplished the first total synthesis of (+)-isochamaecydin (**26**).

On the other hand, starting from **122** and (−)-**123,** the authors adopted the same procedures as for the synthesis of **125** to obtain the pentacyclic product **126**, which underwent the same functional group transformations for the synthesis of (+)-**26** to complete the first total synthesis of (+)-chamaecydin (**27**). The successful collective total synthesis of the two structurally complex natural products demonstrated the high efficiency of the modular synthetic strategy based on the desymmetric enantioselective reduction of cyclic 1,3-dicarbonyl compounds.

## Summary and Outlook

In this review, we summarized the application of desymmetric enantioselective reduction of cyclic 1,3-dicarbonyl compounds in the total synthesis of a series of terpenoid and alkaloid natural products reported between 2016 and 2025. It was evident that the application frequency of this strategy in the synthesis of terpenoid and alkaloid natural products has increased significantly over these ten years compared to the period before 2016. Notably, the substrates of desymmetric enantioselective reduction were no longer limited to monocyclic 1,3-dicarbonyl compounds but could also be extended to bicyclic dicarbonyl compounds. The diversification of substrates enabled this strategy to provide a novel alternative for the synthetic design of structurally more complex natural products. More importantly, from these previous works, it could be observed that the early-stage application of desymmetric enantioselective reduction could efficiently provide access to enantiomerically enriched intermediates with multiple stereocenters containing an all-carbon quaternary chiral center. Taking aforementioned advantages of this strategy, the synthetic efficiency was significantly improved.

Despite remarkable progress has been made, there is still a long way to go before the strategy achieves maturity for applications in natural products. Specifically, challenges remain in improving the enantioselectivity and diastereoselectivity of the desymmetric enantioselective reduction of some complex substrates, as well as suppressing the double reduction by-products and developing new types of catalysts. Moreover, the application of desymmetric enantioselective reduction of chain dicarbonyl compounds in natural products synthesis remains largely undeveloped although several desymmetric enantioselective reduction studies utilizing malonate ester as substrates have been achieved [87–89]. Nevertheless, we believed on the basis of these pioneering works of the development of new reagents and methodologies for desymmetric enantioselective reduction based on the novel dicarbonyl substrates, as well as their applications in the synthesis of various complex natural products will become a focal point in the field of organic synthetic chemistry.

## Data Availability

Data sharing is not applicable as no new data was generated or analyzed in this study.
